# An exploratory study evaluating the predictive capacity of the CALLY index and SOFA-2 for 28-day all-cause mortality in patients with septic shock

**DOI:** 10.3389/fmed.2026.1850768

**Published:** 2026-07-08

**Authors:** Shenglin Su, Ziyue Ma, Qinfu Liu, Zhaojun Wang, Xiaojun Yang

**Affiliations:** 1 Department of Critical Care Medicine, General Hospital of Ningxia Medical University, Yinchuan, Ningxia, China; 2 The First Clinical College of Ningxia Medical University, Yinchuan, Ningxia, China

**Keywords:** CALLY, immunosuppression, sepsis, septic shock, SOFA-2

## Abstract

**Background:**

Early risk stratification of septic shock remains challenging. Although the SOFA score is widely used, its prognostic accuracy is limited in contemporary ICU practice. The C-reactive protein-albumin-lymphocyte (CALLY) index reflects inflammation, nutritional reserve, and immune status, but its independent and incremental prognostic value beyond organ dysfunction scores remains uncertain.

**Methods:**

In this retrospective cohort study, 204 patients with septic shock were enrolled and stratified according to 28-day mortality. Baseline clinical characteristics, laboratory parameters, and severity scores were collected. The prognostic value of CALLY, SOFA, and SOFA-2 was evaluated using univariable and multivariable logistic regression analyses. Discriminative ability was assessed using receiver operating characteristic (ROC) curves, and pairwise AUC comparisons were planned using the DeLong test. In addition, subgroup analyses were performed to explore the stability of associations between CALLY and mortality across predefined clinical subgroups.

**Results:**

Among 204 patients, 104 (51.0%) died within 28 days. Non-survivors presented with older age, higher lactate levels, increased inflammatory markers, worse organ dysfunction, and higher severity scores. In multivariable analysis, SOFA-2 remained independently associated with 28-day mortality (adjusted OR 1.26, 95% CI 1.08–1.50; *p* = 0.005), whereas CALLY was not an independent predictor. ROC analysis showed numerically better discrimination for SOFA-2 than SOFA (AUC 0.679 vs. 0.624), while the addition of CALLY provided only minimal improvement (SOFA-2 + CALLY AUC 0.686 vs. SOFA-2 AUC 0.679). Using individual-level predicted probabilities for reanalysis, the pairwise DeLong test results are shown: SOFA-2 vs. SOFA, *p* = 0.0086; SOFA-2 + CALLY vs. SOFA-2, *p* = 0.3755. Subgroup analysis demonstrated that higher CALLY levels were associated with reduced mortality risk in unadjusted subgroup comparisons, particularly among patients aged > = 65 years (P for interaction = 0.042), but this association was not consistent after adjustment for confounders. Overall, SOFA-2 showed the strongest prognostic signal in this cohort, while the incremental prognostic value of CALLY was limited.

**Conclusion:**

SOFA-2 showed numerically better prognostic performance than conventional SOFA for 28-day mortality in patients with septic shock. Although CALLY was associated with outcomes in descriptive and subgroup analyses, it was not an independent predictor and did not provide a clinically meaningful incremental improvement when added to SOFA-2 in this cohort.

## Background

Sepsis is a life-threatening organ dysfunction caused by a dysregulated host response to infection and remains a major cause of morbidity and mortality among critically ill patients (1) Timely recognition, early intervention, and repeated reassessment are essential for improving outcomes (2). Existing tools used for sepsis assessment and prognostic stratification include the Systemic Inflammatory Response Syndrome (SIRS) criteria, the Simplified Acute Physiology Score II (SAPS II), the Sequential Organ Failure Assessment (SOFA) score, and the Acute Physiology and Chronic Health Evaluation II (APACHE II) score (3–5). The original SOFA score has been widely applied since the 1990s and evaluates neurological, cardiovascular, respiratory, hepatic, renal, and coagulation dysfunction, with higher scores indicating more severe organ dysfunction (6, 7). However, clinical practice, monitoring approaches, and sepsis phenotyping have evolved substantially. Recent discussions have therefore suggested that the SOFA score may require updating to better reflect contemporary ICU practice (8). The SOFA-2 score was subsequently developed and validated to update organ dysfunction assessment while preserving clinical interpretability (9, 10). At the same time, sepsis is highly heterogeneous, and prognosis may also be influenced by inflammatory burden, immune dysfunction, nutritional status, comorbidity burden, infection source, and treatment response (11–14).

The CALLY index incorporates serum albumin, C-reactive protein, and lymphocyte count, representing nutritional status, inflammatory response, and immune function, respectively. Its formula is defined as follows: CALLY = [albumin (g/L) x lymphocyte count (10^9/L)] / [C-reactive protein (mg/L)]. As a composite clinical index, CALLY may provide an integrated signal of inflammatory burden and immunonutritional depletion (15–20). Nevertheless, it does not directly assess organ dysfunction and should not be interpreted as a substitute for organ failure scores. Based on this rationale, the present study explored whether CALLY provides additional prognostic information when evaluated alongside conventional SOFA and SOFA-2 in patients with septic shock.

Therefore, this study aimed to evaluate the predictive performance of SOFA, SOFA-2, and the CALLY index for 28-day all-cause mortality in patients with septic shock, and to determine whether adding CALLY to SOFA- or SOFA-2-based models provides meaningful incremental prognostic value.

## Methods

### Study design and data source

This was a single-center retrospective observational cohort study. The study population comprised patients with septic shock admitted to the Intensive Care Unit (ICU) of the General Hospital of Ningxia Medical University between January 1, 2025, and December 31, 2025. The study was conducted in the hospital’s comprehensive ICU. Data were obtained from the hospital’s electronic medical record system (Hospital Information System, HIS), including admission records, physician order entries, nursing records, laboratory and imaging reports, and clinical progress notes. All data were de-identified after extraction and used solely for research purposes. The study protocol was approved by the Hospital’s Medical Ethics Committee (Approval No. KYLL-2023-0480, Yinchuan, China) and conducted in accordance with the principles of the Declaration of Helsinki. Written informed consent was obtained from all participants.

### Inclusion and exclusion criteria

Inclusion criteria: (1) age > = 18 years; (2) admission to the intensive care unit (ICU) between January 1, 2025, and December 31, 2025; (3) diagnosis of septic shock according to the Sepsis-3 criteria (21), defined as suspected or confirmed infection with an increase in the Sequential Organ Failure Assessment (SOFA) score of > = 2 points from baseline and, despite adequate fluid resuscitation, requirement for vasopressor therapy to maintain a mean arterial pressure (MAP) > =65 mmHg together with serum lactate >2 mmol/L (18 mg/L); and (4) availability of outcome data for the primary analysis (28-day survival status) and key baseline variables.

**Figure 1:**
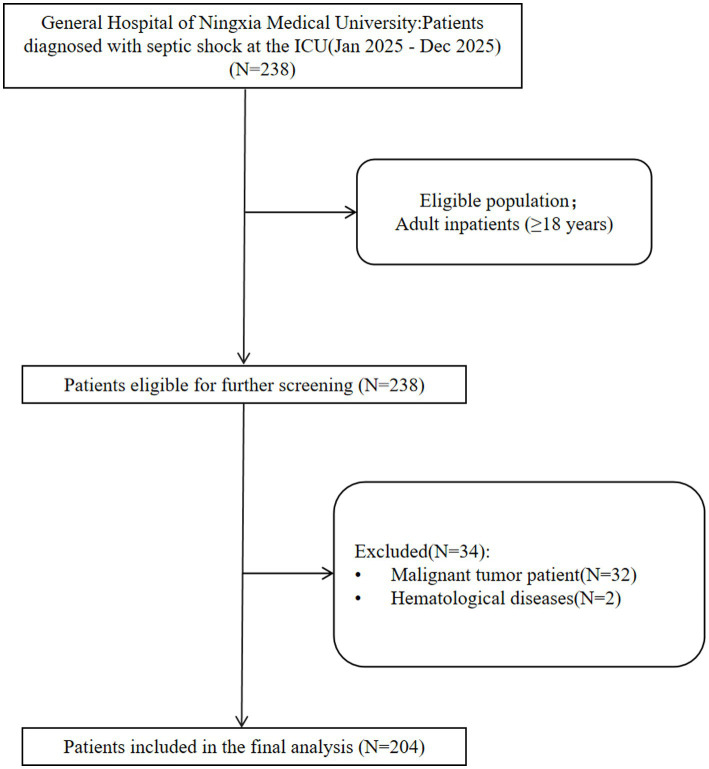
Research population screening process.

Exclusion criteria: (1) active malignancy or hematological disease; (2) missing data for key variables (C-reactive protein [CRP], albumin, lymphocyte count, or outcome/follow-up data); and (3) long-term use of glucocorticoids(Patients who are receiving, or have recently received, systemic glucocorticoid therapy prior to enrollment, at a dose equivalent to prednisone > = 20 mg/day for > = 14 consecutive days (22). or immunosuppressive agents, or pregnancy or breastfeeding. For patients with multiple ICU admissions, only the first admission was included. The study flow diagram is presented in Figure 1.

### Data collection

Baseline data were collected at the time of initial diagnosis and prior to any therapeutic interventions (23). The variables collected included demographic characteristics, comorbidities, infection-related characteristics, and laboratory parameters, specifically age, sex, history of diabetes mellitus, hypertension, cardiovascular disease, smoking history, alcohol consumption, surgery type, causative pathogen, complete blood count, and levels of C-reactive protein (CRP), albumin (ALB), and interleukin-6 (IL-6).

Smoking history was defined as current smoking or a documented history of regular tobacco use before ICU admission. Alcohol consumption was defined as current alcohol intake or a documented history of regular alcohol drinking in the medical record. These variables were extracted from admission records and physician documentation.

The CALLY index was calculated using the following formula: CALLY = ALB (g/L) x lymphocyte count (x10^9/L) / CRP (mg/L)

### Outcome measures

The primary outcome of this study was 28-day survival status following the index time. The index time was defined as the time of confirmed diagnosis of septic shock, specifically the first documented instance of septic shock in the clinical progress notes that met the Sepsis-3 diagnostic criteria. Follow-up commenced at the index time and continued until the earliest of the following events: 28 days after diagnosis or death.

### Statistical analysis

All statistical analyses were performed on the final study cohort after excluding patients with missing data for key variables. Continuous variables were presented as mean ± standard deviation (SD) or median with interquartile range (IQR), as appropriate, according to data distribution. Comparisons between survivors and non-survivors were conducted using Student’s *t* test or the Mann–Whitney *U* test for continuous variables, and the chi-square (χ^2^) test or Fisher’s exact test for categorical variables, as appropriate.

Univariable logistic regression analysis was performed to explore factors associated with 28-day all-cause mortality. Multivariable logistic regression models were constructed to assess the independent prognostic value of the CALLY index, SOFA score, and SOFA-2 score. Covariates were selected *a priori* according to clinical relevance and prior literature on sepsis prognosis rather than solely according to univariable statistical significance, including age, sex, hypertension, diabetes mellitus, coronary heart disease, smoking history, alcohol consumption, type of surgery, causative pathogen, and ejection fraction (16, 18, 24, 25), In addition, to avoid potential overfitting caused by adjustment for an excessive number of covariates, we further performed a sensitivity analysis using a parsimonious model that included only Age, Gender, Hypertension, Diabetes, and CHD. This analysis was conducted to further verify the stability and robustness of the main findings under a reduced adjustment framework. Because APACHE II overlaps conceptually and at the item level with SOFA and SOFA-2, models including APACHE II were interpreted with caution, potential collinearity was acknowledged in the limitations, and sensitivity analyses excluding APACHE II were performed. Results were reported as odds ratios (ORs) or adjusted odds ratios (aORs) with 95% confidence intervals (CIs).

Receiver operating characteristic (ROC) curve analysis was performed to evaluate and compare the predictive performance of SOFA, SOFA-2, SOFA combined with CALLY, and SOFA-2 combined with CALLY for 28-day mortality. The area under the curve (AUC) was calculated for each model, and the optimal cutoff value was determined using the Youden index. Pairwise comparisons of AUCs were performed using the DeLong test. In addition, exploratory subgroup analyses were conducted to examine the association between the CALLY index and 28-day mortality across predefined subgroups. Interaction tests were used to assess potential heterogeneity in this association among different subgroups. All statistical tests were two-sided, and a *p* value <0.05 was considered statistically significant.

## Results

### Patient baseline characteristics

In this cohort of 204 patients with septic shock, 100 survived and 104 did not. Compared with survivors, non-survivors were significantly older (65.61 ± 15.51 vs. 58.72 ± 15.09 years, *p* = 0.002) and had higher heart rates (118.45 ± 27.62 vs. 108.31 ± 25.02 bpm, *p* = 0.007), while cardiac function as assessed by ejection fraction was lower (56.67 ± 11.55 vs. 60.83 ± 8.07%, *p* = 0.003). Markers of tissue hypoperfusion and inflammation were more pronounced in non-survivors, including elevated lactate (4.63 ± 5.18 vs. 2.24 ± 2.04 mmol/L, *p* < 0.001), higher white blood cell and neutrophil counts (*p* = 0.019 and *p* = 0.022, respectively), and increased CRP levels (182.05 [88.88–249.40] vs. 95.00 [54.25–194.43]mg/L, *p* < 0.001). Coagulation dysfunction was also more severe in non-survivors, as reflected by prolonged prothrombin time (15.82 ± 4.72 vs. 13.91 ± 4.59 s, *p* = 0.004). In addition, renal function (BUN: 14.18 ± 9.02 vs. 11.76 ± 7.41 mmol/L, *p* = 0.038) and cardiac stress markers (pro-BNP: 10145.20 ± 10330.44 vs. 6150.03 ± 8784.10 pg./mL, *p* = 0.003) were significantly worse in non-survivors. Severity scores were consistently higher in the non-survival group, including APACHE II, SOFA, and SOFA-2 (all P < = 0.002), indicating greater overall illness severity. Notably, non-survivors required more intensive organ support, with higher rates of mechanical ventilation (89.42% vs. 69.00%, *p* < 0.001), renal replacement therapy (52.88% vs. 24.00%, *p* < 0.001), and vasopressor use (*p* = 0.006). Furthermore, the CALLY index was significantly lower in non-survivors (0.13 [0.06–0.29] vs. 0.23 [0.11–0.76], *p* < 0.001), suggesting its potential association with poor prognosis. No significant differences were observed in sex, comorbidities, infection etiology, or most biochemical parameters between the two groups (*p* > 0.05). These findings indicate that non-survivors exhibited more severe systemic inflammation, organ dysfunction, and hemodynamic instability at baseline (Table 1).

**Table 1:** Baseline characteristics of 204 patients with septic shock.

Variables	All patients (*n* = 204)	Survival (*n* = 100)	Non-survival (*n* = 104)	*p*
Age (years), Mean ± SD	62.23 ± 15.65	58.72 ± 15.09	65.61 ± 15.51	**0.002**
Sex (female), n(%)	66 (32.35)	31 (31.00)	35 (33.65)	0.685
Diabetes (Yes), n (%)	58 (28.43)	32 (32.00)	26 (25.00)	0.268
Hypertension (Yes), n (%)	93 (45.59)	42 (42.00)	51 (49.04)	0.313
Cardiovascular disease (Yes), n (%)	42 (20.59)	17 (17.00)	25 (24.04)	0.214
Smoking history (Yes), n (%)	39 (19.12)	14 (14.00)	25 (24.04)	0.068
Alcohol consumption (Yes), n (%)	27 (13.24)	12 (12.00)	15 (14.42)	0.610
MAP (mmhg), Mean ± SD	79.41 ± 18.93	80.72 ± 18.47	78.15 ± 19.36	0.334
Temperature (°C), Mean ± SD	38.99 ± 1.14	38.97 ± 1.05	39.01 ± 1.24	0.786
Heart Rate (per minute), Mean ± SD	113.48 ± 26.80	108.31 ± 25.02	118.45 ± 27.62	**0.007**
Respiratory Rate (per minute), Mean ± SD	21.48 ± 7.37	21.13 ± 7.68	21.81 ± 7.08	0.513
GCS, Mean ± SD	13.26 ± 2.72	13.33 ± 2.50	13.19 ± 2.93	0.719
EF%, Mean ± SD	58.71 ± 10.19	60.83 ± 8.07	56.67 ± 11.55	**0.003**
PH, Mean ± SD	7.34 ± 0.12	7.34 ± 0.11	7.33 ± 0.13	0.674
SpO2 (mmhg), Mean ± SD	101.70 ± 48.03	100.69 ± 42.64	102.67 ± 52.88	0.769
Lac (mmol/L), Mean ± SD	3.46 ± 4.14	2.24 ± 2.04	4.63 ± 5.18	**<0.001**
BE (mmol/L), Mean ± SD	−5.22 ± 7.64	−4.93 ± 7.33	−5.49 ± 7.95	0.602
WBC (x10^9/L), Mean ± SD	14.90 ± 8.89	13.42 ± 7.39	16.32 ± 9.96	**0.019**
NEUT (x10^9/L), Mean ± SD	12.97 ± 8.12	11.65 ± 6.84	14.24 ± 9.03	**0.022**
HGB (g/L), Mean ± SD	111.63 ± 29.84	114.03 ± 27.66	109.33 ± 31.76	0.262
PLT (x10^9/L), Mean ± SD	158.24 ± 101.25	173.57 ± 101.61	143.49 ± 99.16	**0.034**
K^+^ (mmol/L), Mean ± SD	3.87 ± 0.70	3.83 ± 0.67	3.90 ± 0.74	0.496
Na^+^ (mmol/L), Mean ± SD	141.46 ± 8.08	140.40 ± 7.38	142.48 ± 8.61	0.066
BUN (mmol/L), Mean ± SD	12.99 ± 8.34	11.76 ± 7.41	14.18 ± 9.02	**0.038**
CR (umol/L), Mean ± SD	147.22 ± 112.43	140.83 ± 116.84	153.37 ± 108.23	0.427
AST (U/L), Mean ± SD	227.90 ± 675.54	169.28 ± 538.71	284.26 ± 783.55	0.225
ALT (U/L), Mean ± SD	169.86 ± 445.41	147.07 ± 415.72	191.78 ± 473.18	0.475
PT(s), Mean ± SD	14.89 ± 4.74	13.91 ± 4.59	15.82 ± 4.72	**0.004**
APTT(s), Mean ± SD	40.80 ± 29.17	37.07 ± 14.03	44.40 ± 38.22	0.073
D-dimer (ug/ml FEU), Mean ± SD	9.95 ± 12.98	8.94 ± 13.09	10.92 ± 12.86	0.279
TnI (ng/ml), Mean ± SD	2.04 ± 8.62	1.24 ± 7.66	2.80 ± 9.42	0.196
Pro-BNP (pg/ml), Mean ± SD	8186.78 ± 9787.03	6150.03 ± 8784.10	10145.20 ± 10330.44	**0.003**
PCT (ng/ml), Mean ± SD	27.03 ± 31.55	24.70 ± 30.22	29.28 ± 32.77	0.301
Mechanical ventilation (Yes), n(%)	162 (79.41)	69 (69.00)	93 (89.42)	**<0.001**
RRT, n(%)	79 (38.73)	24 (24.00)	55 (52.88)	**<0.001**
APACHE II, Mean ± SD	17.69 ± 5.51	16.47 ± 5.69	18.87 ± 5.08	**0.002**
SOFA, Mean ± SD	10.08 ± 2.94	9.42 ± 2.92	10.72 ± 2.82	**0.001**
SOFA-2, Mean ± SD	9.06 ± 3.96	7.83 ± 3.90	10.24 ± 3.66	**<0.001**
LOS-ICU, Mean ± SD	10.62 ± 12.21	11.61 ± 9.57	9.66 ± 14.28	0.256
LYM (x10^9//L), M (Q1, Q3)	0.84 (0.45, 1.40)	0.88 (0.57, 1.41)	0.72 (0.40, 1.40)	0.100
ALB (g/L), M (Q1, Q3)	28.02 (25.34, 30.59)	28.05 (25.58, 30.84)	27.96 (25.27, 30.22)	0.760
CRP (mg/L), M (Q1, Q3)	134.65 (58.45, 216.49)	95.00 (54.25, 194.43)	182.05 (88.88, 249.40)	**<0.001**
CALLY, M (Q1, Q3)	0.18 (0.08, 0.50)	0.23 (0.11, 0.76)	0.13 (0.06, 0.29)	**<0.001**
Surgery, n(%)				0.380
NO	125 (61.27)	60 (60.00)	65 (62.50)	
Selective operation	21 (10.29)	8 (8.00)	13 (12.50)	
Emergency operation	58 (28.43)	32 (32.00)	26 (25.00)	
Etiology, n(%)				0.484
Gram negative bacteria	119 (58.33)	58 (58.00)	61 (58.65)	
Gram positive bacteria	45 (22.06)	25 (25.00)	20 (19.23)	
Fungus	40 (19.61)	17 (17.00)	23 (22.12)	
Vasopressor, n(%)				**0.006**
No	34 (16.67)	25 (25.00)	9 (8.65)	
Yes	159 (77.94)	71 (71.00)	88 (84.62)	
Combination	11 (5.39)	4 (4.00)	7 (6.73)	

SD, standard deviation; M, Median; Q1, 1st Quartile; Q3, 3rd Quartile; MAP, Mean Arterial Pressure; GCS, Glasgow Coma Scale; EF, Ejection Fraction; PH, Potential of Hydrogen; SpO2, Peripheral Capillary Oxygen Saturation; Lac, Lactate; BE, Base Excess; WBC, White Blood Cell Count; NEUT, Neutrophil Count; HGB, Hemoglobin; PLT, Platelet Count; BUN, Blood Urea Nitrogen; CR, Creatinine; AST, Aspartate Aminotransferase; ALT, Alanine Aminotransferase; PT, Prothrombin Time; APTT, Activated Partial Thromboplastin Time; TnI, Troponin I; Pro-BNP, Pro–B-type Natriuretic Peptide; PCT, Procalcitonin; RRT, Renal Replacement Therapy; APACHE II, Acute Physiology and Chronic Health Evaluation II; SOFA, Sequential Organ Failure Assessment; LOS-ICU, Length of Stay in Intensive Care Unit; LYM, Lymphocyte Count; ALB, Albumin; CRP, C-reactive Protein; CALLY, CALLY Index, (CRP-Albumin-Lymphocyte Index); ICU, Intensive Care Unit. Bold values indicate statistical significance (*p* < 0.05).

### Single factor analysis

In univariate logistic regression analysis, older age (OR 1.03, 95% CI 1.01–1.05; *p* = 0.002), higher heart rate (OR 1.01, 95% CI 1.01–1.03; *p* = 0.008), lactate (OR 1.25, 95% CI 1.10–1.42; *p* < 0.001), white blood cell count (OR 1.04, 95% CI 1.01–1.08; *p* = 0.022), neutrophil count (OR 1.04, 95% CI 1.01–1.08; *p* = 0.025), blood urea nitrogen (OR 1.04, 95% CI 1.01–1.07; *p* = 0.041), prothrombin time (OR 1.11, 95% CI 1.03–1.19; *p* = 0.006), pro-BNP (OR 1.01, 95% CI 1.01–1.01; *p* = 0.004), CRP (OR 1.01, 95% CI 1.01–1.01; *p* < 0.001), mechanical ventilation (OR 3.80, 95% CI 1.79–8.08; *p* < 0.001), renal replacement therapy (OR 3.55, 95% CI 1.95–6.47; *p* < 0.001), vasopressor use (OR 3.44, 95% CI 1.51–7.84; *p* = 0.003) or combination vasopressor therapy (OR 4.86, 95% CI 1.15–20.63; *p* = 0.032), as well as higher APACHE II, SOFA, and SOFA-2 scores (OR 1.09, 95% CI 1.03–1.14; *p* = 0.002; OR 1.17, 95% CI 1.06–1.29; *p* = 0.002; and OR 1.18, 95% CI 1.09–1.28; *p* < 0.001, respectively), were significantly associated with increased odds of non-survival. In contrast, higher ejection fraction (OR 0.96, 95% CI 0.93–0.99; *p* = 0.005) and platelet count (OR 0.99, 95% CI 0.99–0.99; *p* = 0.036) were associated with lower odds of non-survival. No significant associations were observed for sex, major comorbidities, surgical status, infection etiology, or CALLY (OR 0.84, 95% CI 0.65–1.09; *p* = 0.188). Notably, the effects of biomarkers such as pro-BNP and CRP should be interpreted on a per-unit increase basis according to their measurement scales (Table 2).

**Table 2:** The results of univariate logistic regression for septic shock.

Variables	*β*	S. E	Z	*p*	OR (95% CI)
Age (years)	0.03	0.01	3.08	**0.002**	1.03 (1.01–1.05)
Sex (female)	−0.12	0.30	−0.40	0.686	0.89 (0.49–1.59)
Diabetes (Yes)	−0.34	0.31	−1.11	0.269	0.71 (0.38–1.31)
Hypertension (Yes)	0.28	0.28	1.01	0.313	1.33 (0.76–2.31)
Cardiovascular disease (Yes)	0.44	0.35	1.24	0.216	1.55 (0.78–3.08)
Smoking history (Yes)	0.66	0.37	1.80	0.071	1.94 (0.94–4.00)
Alcohol consumption (Yes)	0.21	0.42	0.51	0.610	1.24 (0.55–2.79)
MAP (mmhg)	−0.01	0.01	−0.97	0.333	0.99 (0.98–1.01)
Temperature (°C)	0.03	0.12	0.27	0.785	1.03 (0.81–1.32)
Heart Rate (per minute)	0.01	0.01	2.67	**0.008**	1.01 (1.01–1.03)
Respiratory Rate (per minute)	0.01	0.02	0.66	0.511	1.01 (0.98–1.05)
GCS	−0.02	0.05	−0.36	0.717	0.98 (0.89–1.09)
EF%	−0.04	0.02	−2.82	**0.005**	0.96 (0.93–0.99)
PH	−0.50	1.17	−0.42	0.672	0.61 (0.06–6.06)
SpO2 (mmhg)	0.00	0.00	0.29	0.768	1.00 (1.00–1.01)
Lac (mmol/L)	0.23	0.06	3.51	**<0.001**	1.25 (1.10–1.42)
BE (mmol/L)	−0.01	0.02	−0.52	0.601	0.99 (0.96–1.03)
WBC (x10^9/L)	0.04	0.02	2.28	**0.022**	1.04 (1.01–1.08)
NEUT (x10^9/L),	0.04	0.02	2.24	**0.025**	1.04 (1.01–1.08)
HGB (g/L)	−0.01	0.00	−1.12	0.261	0.99 (0.99–1.00)
PLT (x10^9/L)	−0.01	0.00	−2.10	**0.036**	0.99 (0.99–0.99)
K^+^(mmol/L)	0.14	0.20	0.68	0.494	1.15 (0.77–1.70)
Na^+^(mmol/L)	0.03	0.02	1.82	0.069	1.03 (1.00–1.07)
BUN (mmol/L)	0.04	0.02	2.05	**0.041**	1.04 (1.01–1.07)
CR (umol/L)	0.00	0.00	0.80	0.426	1.00 (1.00–1.00)
AST (U/L)	0.00	0.00	1.15	0.249	1.00 (1.00–1.00)
ALT (U/L)	0.00	0.00	0.71	0.477	1.00 (1.00–1.00)
PT(s)	0.10	0.04	2.76	**0.006**	1.11 (1.03–1.19)
APTT(s)	0.02	0.01	1.84	0.065	1.02 (1.00–1.04)
D-dimer (ug/ml FEU)	0.01	0.01	1.07	0.285	1.01 (0.99–1.04)
TnI (ng/ml)	0.02	0.02	1.21	0.226	1.03 (0.98–1.07)
Pro-BNP (pg/ml)	0.01	0.00	2.85	**0.004**	1.01 (1.01–1.01)
PCT (ng/ml)	0.00	0.00	1.04	0.300	1.00 (1.00–1.01)
Mechanical ventilation (Yes)	1.33	0.39	3.46	**<0.001**	3.80 (1.79–8.08)
RRT	1.27	0.31	4.15	**<0.001**	3.55 (1.95–6.47)
APACHE II	0.08	0.03	3.05	**0.002**	1.09 (1.03–1.14)
SOFA	0.16	0.05	3.11	**0.002**	1.17 (1.06–1.29)
SOFA-2	0.17	0.04	4.20	**<0.001**	1.18 (1.09–1.28)
LOS-ICU	−0.01	0.01	−1.12	0.264	0.99 (0.96–1.01)
LYM (x10^9/L)	0.04	0.13	0.29	0.770	1.04 (0.80–1.35)
ALB (g/L)	0.00	0.03	0.14	0.889	1.00 (0.95–1.06)
CRP (mg/L)	0.01	0.00	3.70	**<0.001**	1.01 (1.01–1.01)
CALLY	−0.17	0.13	−1.32	0.188	0.84 (0.65–1.09)
Surgery					
NO					1.00 (Reference)
Selective operation	0.41	0.48	0.84	0.402	1.50 (0.58–3.87)
Emergency operation	−0.29	0.32	−0.90	0.367	0.75 (0.40–1.40)
Etiology					
Gram negative bacteria					1.00 (Reference)
Gram positive bacteria	−0.27	0.35	−0.78	0.437	0.76 (0.38–1.52)
Fungus	0.25	0.37	0.68	0.495	1.29 (0.62–2.65)
Vasopressor					
No					1.00 (Reference)
Yes	1.24	0.42	2.94	**0.003**	3.44 (1.51–7.84)
Combination	1.58	0.74	2.14	**0.032**	4.86 (1.15–20.63)

OR, Odds Ratio; CI, Confidence Interval. Bold values indicate statistical significance (*p* < 0.05).

### Prediction value of SOFA-2 for 28-day all-cause mortality in patients with septic shock

**Figure 2:**
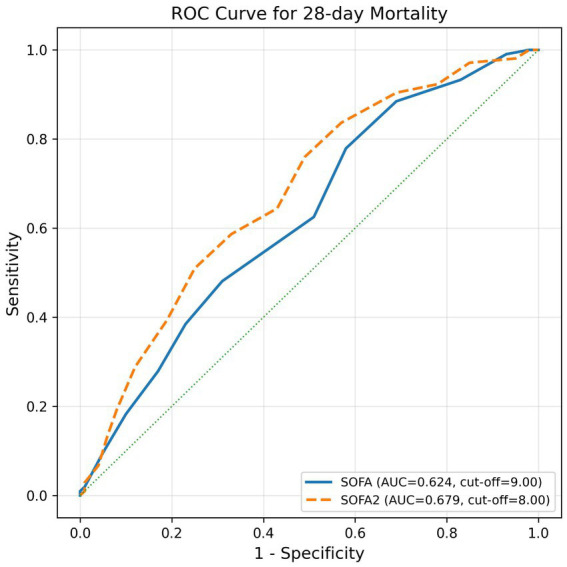
Comparison of the predictive value of SOFA and SOFA-2 for 28-day mortality in septic shock.

**Figure 3:**
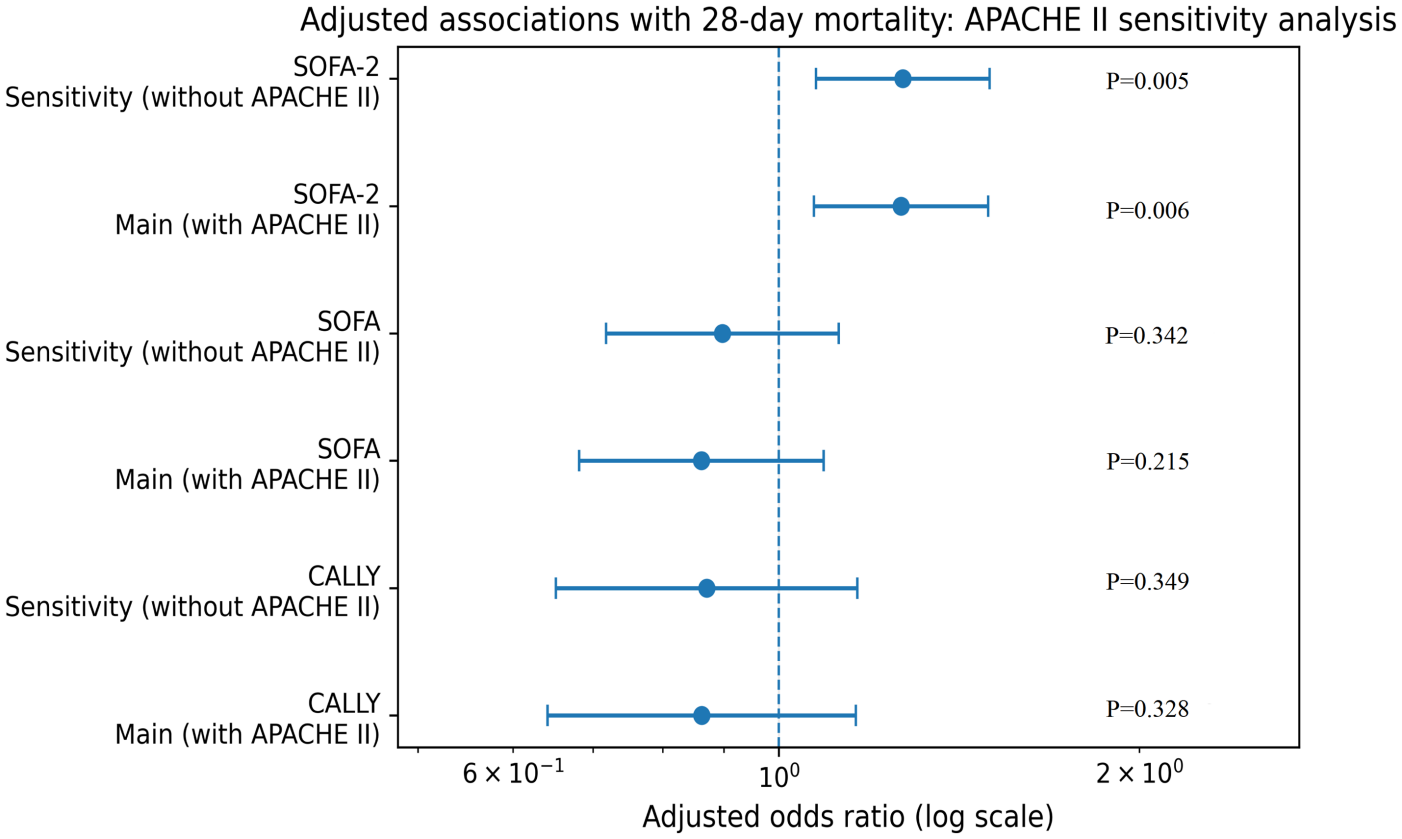
APACHE II sensitivity analysis of adjusted associations with 28-day mortality.

In logistic regression analyses of 28-day mortality (Table 3), CALLY was not significantly associated with the outcome in either the univariable or adjusted model (OR 0.84, 95% CI 0.64–1.07; *p* = 0.188; adjusted OR 0.87, 95% CI 0.65–1.15; *p* = 0.349). SOFA was significantly associated with 28-day mortality in univariable analysis (OR 1.17, 95% CI 1.06–1.30; *p* = 0.002), but this association was no longer significant after adjustment for confounders (adjusted OR 0.85, 95% CI 0.67–1.08; *p* = 0.186). In contrast, SOFA-2 remained independently associated with 28-day mortality in both univariable and multivariable analyses (OR 1.18, 95% CI 1.10–1.28; *p* < 0.001; adjusted OR 1.26, 95% CI 1.08–1.50; *p* = 0.005), indicating that each 1-point increase in SOFA-2 was associated with a 26% increase in the odds of death. ROC analysis (Figure 2) showed numerically improved, although still modest, discrimination for SOFA-2 compared with SOFA, with an AUC of 0.679 and a cut-off value of 8.0, versus an AUC of 0.624 and a cut-off value of 9.0 for SOFA. Because APACHE II shares some overlapping variables with SOFA/SOFA-2, potential collinearity or overadjustment may occur; therefore, a sensitivity analysis excluding APACHE II was performed, as shown in Figure 3. Taken together, these results suggest that SOFA-2 may provide better prognostic discrimination for 28-day mortality than conventional SOFA in this cohort, whereas CALLY did not demonstrate independent predictive value.

**Table 3:** Logistic regression analysis of 28-day mortality.

Variable	Univariable		Multivariable	
	OR (95% CI)	*p* value	aOR (95% CI)	*p* value
CALLY	0.84 (0.64–1.07)	0.188	0.87 (0.65–1.15)	0.349
SOFA	1.17 (1.06–1.30)	**0.002**	0.85 (0.67–1.08)	0.186
SOFA-2	1.18 (1.10–1.28)	**<0.001**	1.26 (1.08–1.50)	**0.005**

Multivariable model adjusted for age, sex, hypertension, diabetes mellitus, coronary heart disease, smoking history, alcohol consumption, surgery type, causative pathogen, ejection fraction, and APACHE II. Covariates were selected a priori based on clinical relevance and prior literature. Bold values indicate statistical significance (*p* < 0.05).

### Predictive value of SOFA, SOFA-2 combined with CALLY for 28-day all-cause mortality in patients with septic shock

**Figure 4:**
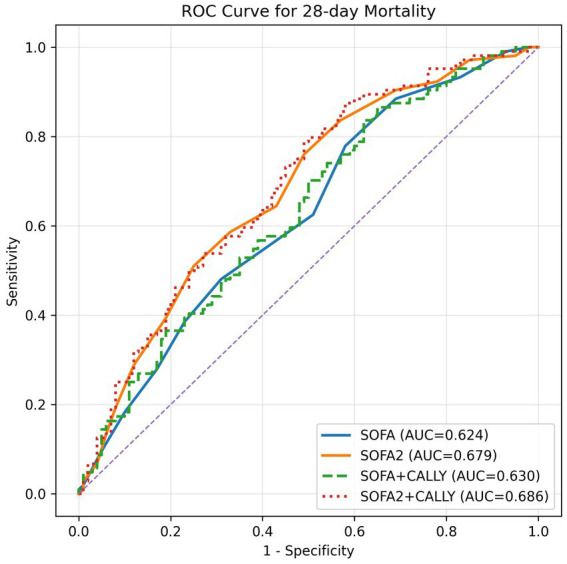
Comparison of the predictive value of SOFA, SOFA-2, and CALLY for 28-day all-cause mortality in septic shock.

**Figure 5:**
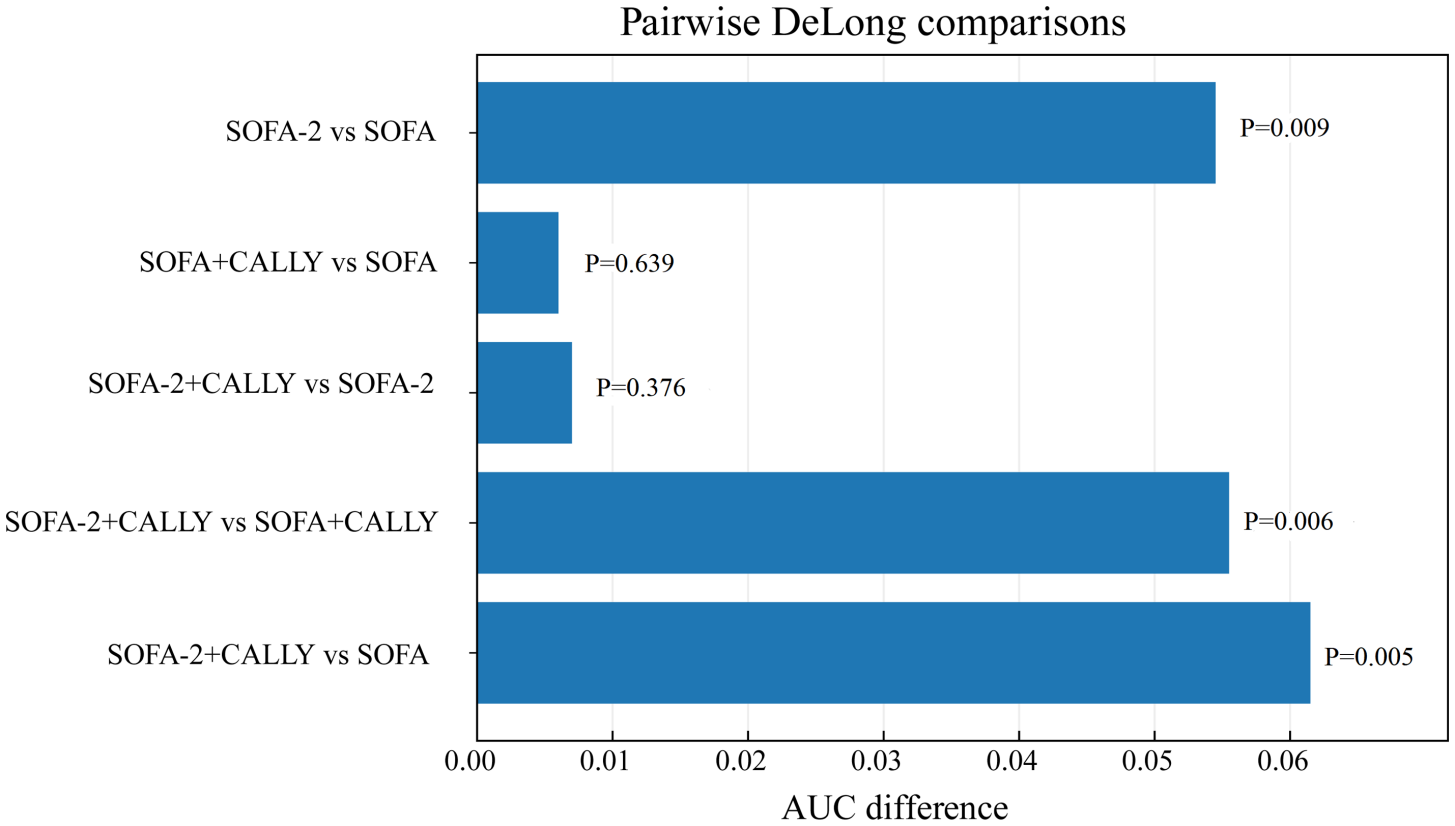
Pairwise DeLong comparisons of AUC values among prediction models.

In the ROC analysis for 28-day mortality (Figure 4), the SOFA-2 + CALLY model yielded the highest AUC numerically (0.686), followed by SOFA-2 alone (AUC 0.679), SOFA+CALLY (AUC 0.630), and SOFA alone (AUC 0.624). However, the incremental increase after adding CALLY was small for both SOFA-based models (from 0.624 to 0.630 for SOFA and from 0.679 to 0.686 for SOFA-2). Pairwise DeLong test results should be reported as follows (Figure 5): SOFA-2 vs. SOFA, *p* = 0.009; SOFA-2 + CALLY vs. SOFA-2, *p* = 0.376 SOFA+CALLY vs. SOFA, *p* = 0.639, SOFA-2 showed a significantly higher AUC than SOFA, whereas adding CALLY to SOFA or SOFA-2 resulted in only small, non-significant AUC improvements. Overall, all AUC values remained below 0.70, suggesting only modest predictive accuracy for 28-day mortality in this cohort. These findings indicate that SOFA-2-based models may offer better numerical prognostic performance than conventional SOFA, while the incremental contribution of CALLY appears limited and should not be overinterpreted.

### Exploratory subgroup analysis of the CALLY index in patients with septic shock

**Figure 6:**
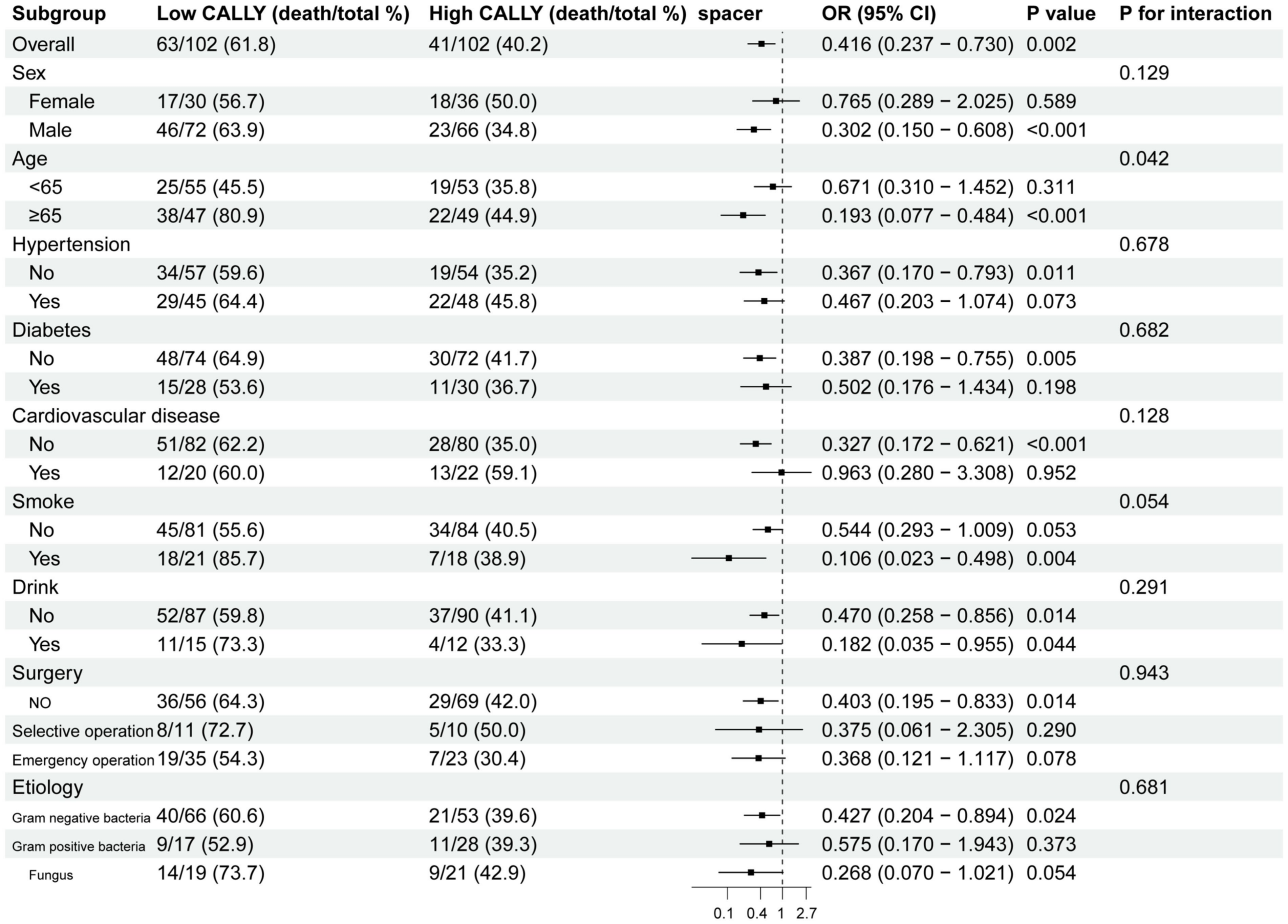
Exploratory subgroup analysis of the CALLY index in patients with septic shock.

Subgroup analysis (Figure 6) suggested that patients with high CALLY had lower 28-day mortality in unadjusted subgroup comparisons than those with low CALLY (OR 0.416, 95% CI 0.237–0.730). This association was generally similar across sex, hypertension, diabetes, coronary heart disease, surgical status, and infection etiology, with no significant interactions observed (P for interaction >0.05). A significant interaction was detected for age (P for interaction = 0.042), with a stronger association observed in patients aged > = 65 years (OR 0.193, 95% CI 0.077–0.484) than in those <65 years (OR 0.671, 95% CI 0.310–1.452). However, because CALLY was not an independent predictor in the adjusted regression model, these subgroup findings should be considered exploratory and hypothesis-generating.

## Discussion

This study evaluated the prognostic performance of SOFA, SOFA-2, and the CALLY index for 28-day all-cause mortality in patients with septic shock. The principal finding was that SOFA-2 remained independently associated with 28-day mortality after adjustment for potential confounders, whereas the CALLY index did not. Although adding CALLY to SOFA-2 produced the highest AUC numerically, the absolute improvement was very small (0.679 to 0.686) and therefore should be interpreted as marginal rather than as evidence of clinically meaningful improvement.

Thus, our findings support the prognostic relevance of updated organ dysfunction assessment in septic shock. They do not establish CALLY as an independent prognostic marker in this cohort. Instead, the CALLY-related findings should be viewed as exploratory and may help generate hypotheses about host-response heterogeneity that require confirmation in larger prospective datasets.

### SOFA-2 and prognostic assessment of sepsis

Sepsis-3 defines sepsis as life-threatening organ dysfunction caused by infection and uses an increase of > = 2 points in the SOFA score as a clinical operational criterion, thereby placing organ function assessment at the core of sepsis identification and stratification (21). The traditional SOFA score has been widely used, but ICU practice and data availability have evolved over recent decades. Recent work has therefore proposed SOFA-2 as an updated approach to organ dysfunction assessment that is more aligned with contemporary ICU data while retaining clinical interpretability (8–10). However, recent studies have found that updating from SOFA-1 to SOFA-2 results in comparable score distributions and maintains the discriminatory capacity for predicting ICU mortality in patients with sepsis (26). In the present cohort, SOFA-2 showed a stronger association with 28-day mortality than conventional SOFA, consistent with the central role of organ dysfunction in septic shock prognosis. Nevertheless, the observed AUCs were modest, and the difference between SOFA and SOFA-2 should be interpreted in light of formal AUC comparison and external validation. Future studies should further evaluate whether dynamic changes in SOFA-2, rather than a single baseline value, improve prognostic discrimination and clinical usefulness (12, 27).

### Potential clinical value of dynamic changes in the CALLY index in septic shock

The CALLY index is composed of C-reactive protein (CRP), albumin, and lymphocyte count and is intended to summarize inflammatory burden, nutritional reserve, and immune status using routinely available laboratory parameters (15, 16, 20). These biological dimensions are relevant to sepsis pathophysiology: CRP reflects systemic inflammation, albumin may reflect nutritional reserve as well as capillary leakage and hepatic synthetic function, and lymphocytopenia may accompany sepsis-related immune dysfunction. However, in the present study, CALLY was not independently associated with 28-day mortality after adjustment. This differs from some previous reports in broader sepsis populations or emergency department cohorts in which CALLY was associated with short-term mortality (16, 20). Recent articles suggest that immune dysregulation in sepsis may be too complex to be fully captured by simple baseline markers. Recent articles suggest that immune dysregulation in sepsis may be too complex to be fully captured by simple baseline markers (26). Potential explanations include differences in patient severity, septic shock-specific pathophysiology, timing of laboratory measurement, endpoint definition, and residual confounding. In early septic shock, intense inflammatory activation, hypoalbuminemia related to acute capillary leakage, and lymphocyte depletion may reduce the stability of a single baseline CALLY measurement. Therefore, our data suggest that baseline CALLY alone has limited prognostic value in septic shock, although serial changes may still merit future investigation.

### Complementary roles of SOFA-2 and CALLY in explaining sepsis heterogeneity

Sepsis is clinically heterogeneous, and risk may vary according to organ dysfunction, infection source, comorbidity burden, immune status, and treatment response (12, 13). SOFA-2 primarily reflects physiological decompensation at the organ-system level, whereas CALLY represents a simplified host-response signal. Conceptually, these dimensions could be complementary. However, the present data show only a minimal numerical increase in AUC after adding CALLY to SOFA-2 and no independent association between CALLY and mortality in the adjusted model. Accordingly, the combined SOFA-2 + CALLY model should be regarded as exploratory rather than ready for clinical implementation. In the future, the CALLY index may prove beneficial for improving clinical sepsis risk stratification after validation in larger dynamic retrospective and prospective cohorts, external validation, calibration assessment, and decision-curve analysis.

### Clinical implications: a practical and scalable tool for early risk stratification

From a clinical perspective, SOFA-2 may be useful for early risk stratification because it updates organ dysfunction assessment while maintaining interpretability. CALLY is inexpensive and easy to calculate, but in this cohort its incremental value beyond SOFA-2 was limited. Therefore, CALLY should not be used as a stand-alone prognostic tool or as a replacement for organ dysfunction scores in septic shock. At most, it may be considered an exploratory adjunct that requires prospective validation, particularly if serial measurements can capture recovery or deterioration in inflammatory, nutritional, and immune status.

### Limitations

This study has several limitations. First, the single-center retrospective design may introduce selection bias and information bias, and some potential confounding factors, including timeliness of source control, antimicrobial therapy, fluid resuscitation, and vasopressor titration, may not have been fully accounted for. Second, the timing and frequency of SOFA-2 and CALLY measurements may vary in real-world practice, and this study mainly assessed baseline values rather than dynamic trajectories. Third, the modest AUC values indicate limited discrimination, and the small numerical gain after adding CALLY should be interpreted cautiously. Fourth, APACHE II overlaps conceptually with SOFA and SOFA-2 because all include acute physiological or organ dysfunction information; therefore, adjustment involving APACHE II may introduce collinearity or overadjustment. Fifth, this study focused on 28-day mortality and did not assess long-term survival, organ function recovery, or quality of life. In addition, patients with missing key variables required for calculating the CALLY index and SOFA2 score were excluded during the cohort selection process. Although this approach ensured the completeness of the analytic dataset, it may have resulted in differences between the final analytic cohort and the original target population, thereby introducing potential selection bias. Future multicenter prospective studies should validate SOFA-2 and CALLY across different infection sources, age groups, and comorbidity profiles; compare dynamic SOFA-2 and serial CALLY trajectories; and evaluate calibration, net clinical benefit, and feasibility in clinical decision-making.

## Conclusion

SOFA-2 showed numerically better prognostic performance than conventional SOFA and remained independently associated with 28-day mortality in patients with septic shock. In contrast, baseline CALLY was not an independent predictor and added only minimal incremental discrimination to SOFA-2 in this cohort. These findings support SOFA-2 as the more robust prognostic measure in this dataset, while the role of CALLY should be considered exploratory and requires validation using prospective, multicenter, and dynamic measurement designs.

## Data Availability

The original contributions presented in the study are included in the article/Supplementary material, further inquiries can be directed to the corresponding author.
