# Supplementary material for: An exploratory study evaluating the predictive capacity of the CALLY index and SOFA-2 for 28-day all-cause mortality in patients with septic shock
Source: Front Med (Lausanne). 2026 Jul 8;13:1850768. doi: 10.3389/fmed.2026.1850768 (PMC13388888; doi:10.3389/fmed.2026.1850768)
Supplement: Supplementary file 1 [file Table_1.DOCX]

**Table S1. Pearson correlation matrix**

| **Score** | **APACHE II** | **SOFA** | **SOFA**-**2** |
| --- | --- | --- | --- |
| **APACHE II** | 1.000 | 0.426 | 0.403 |
| **SOFA** | 0.426 | 1.000 | 0.865 |
| **SOFA**-**2** | 0.403 | 0.865 | 1.000 |

Data are Pearson correlation coefficients. APACHE=Acute Physiology and Chronic Health Evaluation; SOFA=Sequential Organ Failure Assessment.

**Table S2. Logistic regression analysis of 28-day mortality**

| Variable | Univariable | |  | Multivariable | |
| --- | --- | --- | --- | --- | --- |
|  | OR (95% CI) | P value |  | aOR (95% CI) | P value |
| CALLY | 0.84 (0.65-1.09) | 0.188 |  | 0.93 (0.72-1.21) | 0.606 |
| SOFA | 1.17 (1.06-1.29) | 0.002 |  | 0.89 (0.72-1.10) | 0.274 |
| SOFA2 | 1.18 (1.09-1.28) | <0.001 |  | 1.28 (1.09-1.51) | 0.002 |

Multivariable model adjusted for Age, Gender, Hypertension, Diabetes, CHD.
